# Increases in Hepatitis C Virus Infection Related to Injection Drug Use Among Persons Aged ≤30 Years — Kentucky, Tennessee, Virginia, and West Virginia, 2006–2012

**Published:** 2015-05-08

**Authors:** Jon E. Zibbell, Kashif Iqbal, Rajiv C. Patel, Anil Suryaprasad, Kathy J. Sanders, Loretta Moore-Moravian, Jamie Serrecchia, Steven Blankenship, John W. Ward, Deborah Holtzman

**Affiliations:** 1 Division of Viral Hepatitis, National Center for HIV/AIDS, Viral Hepatitis, STD, and TB Prevention, CDC; 2 Kentucky Department for Public Health; 3 Tennessee Department of Health; 4 Virginia Department of Health; 5 West Virginia Department of Health

Hepatitis C virus (HCV) infection is the most common blood-borne infection in the United States, with approximately three million persons living with current infection (1). Percutaneous exposure to contaminated blood is the most efficient mode of transmission, and in the United States, injection drug use (IDU) is the primary risk factor for infection. State surveillance reports from the period 2006–2012 reveal a nationwide increase in reported cases of acute HCV infection, with the largest increases occurring east of the Mississippi River, particularly among states in central Appalachia (2). Demographic and behavioral data accompanying these reports show young persons (aged ≤30 years) from nonurban areas contributed to the majority of cases, with about 73% citing IDU as a principal risk factor. To better understand the increase in acute cases of HCV infection and its correlation to IDU, CDC examined surveillance data for acute case reports in conjunction with analyzing drug treatment admissions data from the Treatment Episode Data Set-Admissions (TEDS-A) among persons aged ≤30 years in four states (Kentucky, Tennessee, Virginia, and West Virginia) for the period 2006–2012. During this period, significant increases in cases of acute HCV infection were found among persons in both urban and nonurban areas, with a substantially higher incidence observed each year among persons residing in nonurban areas. During the same period, the proportion of treatment admissions for opioid dependency increased 21.1% in the four states, with a significant increase in the proportion of persons admitted who identified injecting as their main route of drug administration (an increase of 12.6%). Taken together, these increases indicate a geographic intersection among opioid abuse, drug injecting, and HCV infection in central Appalachia and underscore the need for integrated health services in substance abuse treatment settings to prevent HCV infection and ensure that those who are infected receive medical care.

Confirmed cases of acute HCV infection* and associated demographic and risk characteristics were obtained from the National Notifiable Disease Surveillance System (NNDSS) for Kentucky, Tennessee, Virginia, and West Virginia for the period 2006–2012 for persons aged ≤30 years.† Surveillance case reports met the clinical and laboratory markers of confirmed cases of acute HCV infection as defined by CDC/CSTE.§ A case report was classified as “urban” if the person lived in a metropolitan county with ≥50,000 population and as “nonurban” if the person lived in a nonmetropolitan county with <50,000 population.¶ The percentage of cases reported for the period 2006–2012 among persons aged ≤30 years in the four states were examined by demographic and risk characteristics (IDU versus non-IDU) and by urbanicity. In addition, using the number of cases reported through NNDSS as the numerator and the mid-year (July) population estimates for persons aged ≤30 years from U.S. Census Bureau as the denominator, annual incidence rates for the period 2006–2012 were calculated and analyzed by urbanicity. Linear trends in annual incidence were determined by the Spearman correlation trend test and were considered statistically significant at p<0.05.

TEDS-A contains data on admissions to substance abuse treatment facilities in the United States, by year and state, among patients aged ≥12 years.** For each admission, up to three “substances of abuse” with a corresponding route of administration and demographic characteristics might be reported. TEDS-A classifies opioids into three categories: heroin, nonprescription methadone, and opiates and synthetics. For this report, three types of admissions were defined: heroin admission, prescription opioid admission (includes nonprescription methadone and opiates and synthetics), and any opioid admission (includes heroin and prescription opioids). In addition, two types of drug injection were defined: any opioid injection (includes injection of heroin and/or prescription opioids) and nonopioid injection (includes injection of any substance not classified as an opioid [e.g., cocaine]). The annual percentage of patient admissions among persons aged 12–29 years in Kentucky, Tennessee, Virginia, and West Virginia was calculated by type of admission and by drug injection for the period 2006–2012. Denominators for all percentages were the total number of reported treatment admissions for persons aged 12–29 years in that year in the four states. Further, the difference in the percentage of each admission type from 2006 to 2012 was calculated. Significance of a monotonic trend for any-opioid and nonopioid injection was determined by the Mann-Kendall test. Trends were considered statistically significant at p<0.05.

During 2006–2012, a total of 1,377 cases of acute HCV infection were reported to CDC from Kentucky, Tennessee, Virginia, and West Virginia. Of the 1,374 cases with a recorded age and classified as either urban or nonurban, 616 (44.8%) were among persons aged ≤30 years. The median age of persons with acute infection was 25 years in both nonurban (range = 6–30 years) and urban (range = 6–30 years) counties (Table). Of the number of cases in persons aged ≤30 years in nonurban counties, 247 (78.4%) were in non-Hispanic whites, and 156 (49.5%) in males; in urban counties, 249 (82.7%) cases were in non-Hispanic whites, and 155 (51.5%) were in males. Among the 265 (43.0%) cases in both urban and nonurban counties with identified risks for HCV infection, 196 (73.1%) were among persons who reported IDU, with similar percentages by urbanicity (urban = 99 [71.7%], nonurban 95 [74.8%]). During 2006–2012, a significant increase occurred in the incidence of acute HCV infection among young persons in both nonurban (p=0.007) and urban counties (p<0.001) in the four states (Figure 1). However, in each year, incidence was more than twice the rate among persons who resided in nonurban compared with urban areas.

Among all treatment admissions for persons aged 12–29 years in the four states, the change in the proportion of any-opioid admissions increased by 21.1% from 2006 to 2012 (Figure 2). In addition, increases of 16.8% and 7.4% were observed in the proportion of prescription opioid admissions and heroin admissions, respectively. Further, from 2006 to 2012, the proportion of admissions related to any-opioid injection increased by 12.6%, and the proportion of admissions of a patient reporting nonopioid injection increased by 2.1%. Both trends (any-opioid and nonopioid injections) were significant (p<0.05) over the 7-year period (Figure 3).

## Discussion

Surveillance data from four states (Kentucky, Tennessee, Virginia, West Virginia) showed a substantial increase (364%) in the number of cases of acute HCV infection from 2006 to 2012 among persons aged ≤30 years. Those affected were primarily non-Hispanic-white residents from both urban and nonurban areas, with more than double the rate of cases from nonurban areas. Urban and nonurban cases had the same distribution by sex. Among cases with identified risk information, IDU was most commonly reported (73%). Similar increases among persons with analogous demographic characteristics have been reported over the period (2006–2012) in Massachusetts (3), Wisconsin (4) and upstate New York (5).

During this same period, these four states experienced an increase in the number of adolescents and young adults (aged 12–29 years) admitted to substance abuse treatment for opioid dependency (based on criteria of the *Diagnostic and Statistical Manual of Mental Disorders, Fifth Edition*), with prescription opioid abuse accounting for about one third of all treatment admissions (compared with 8.3% of admissions for heroin). However, during 2011–2012, the proportion of heroin admissions increased (from 8.6% to 12.0%) at the same time as the proportion of prescription opioid admissions decreased. This regional increase in heroin use is consistent with national survey reports estimating an increase in first-time heroin use from 90,000 persons in 2006 to 156,000 persons in 2012, with three out of four persons who used heroin and prescription opioids in the past year reporting prescription opioid misuse before initiating heroin, and a doubling of the number of persons reporting heroin dependency from 214,000 in 2002 to 467,000 in 2012 (6). The concomitant increase in the proportion of treatment admissions for prescription opioid abuse, heroin abuse, and the number of admitted patients who report injecting suggests that the increase in acute HCV infections in central Appalachia is highly correlated with the region’s epidemic of prescription opioid abuse (7) and facilitated by an upsurge in the number of persons who inject drugs in these four states. Increases in the incidence of HCV infection have the potential to thwart the nation’s effort to control morbidity and mortality associated with HCV infection, in addition to undermining the U.S. Department of Health and Human Services’ *Action Plan for the Prevention, Care, and Treatment of Viral Hepatitis* (8), which has set reducing HCV infections caused by drug use behaviors as a priority area.

What is already known on this topic?

Data from 2006–2012 reveal a nationwide increase in reported cases of acute hepatitis C virus (HCV) infection, which is an important cause of morbidity and mortality in the United States. Adolescents and young adults (aged ≤30 years) from nonurban areas account for the majority of cases, with approximately 73% citing injection drug use as the principal risk factor.

What is added by this report?

From 2006 to 2012, there were significant increases in cases of acute HCV infections among persons aged ≤30 years in Kentucky, Tennessee, Virginia, and West Virginia. The increasing incidence among nonurban residents was at least double that of urban residents each year. Treatment admissions for opioid dependency increased 21.1% across the four states, with a significant increase in the proportion of persons admitted who report injecting drugs (a 12.6% increase). These increases indicate a strong correlation among opioid abuse, drug injecting, and HCV infection in these four states.

What are the implications for public health practice?

Evidence-based strategies as well as integrated-service provision are urgently needed in drug treatment programs to ensure patients are tested for HCV and persons found to be HCV-infected are linked to care and receive appropriate treatment. These efforts will require further collaboration among federal partners and state and local health departments to better address the syndemic of opioid abuse and HCV infection.

The findings in this report are subject to at least seven limitations. First, the inability to link identified HCV cases to individual treatment admissions makes this analysis ecologic; therefore, the concomitant increase of acute HCV cases and prescription opioid admissions among persons reporting IDU should not be considered causally related. Still, IDU is the primary risk factor for HCV infection in the United States, and 73% of acute case reports with identified risks for HCV infection specify IDU. Second, the current surveillance case definition for acute HCV infection captures only persons with signs and symptoms of illness, and because acute infections are often asymptomatic, the underreporting of cases is likely. Third, because acute hepatitis C incidence by state and county were calculated from passive and voluntary case reporting to NNDSS, these data should not be interpreted as definitive state and county incidence estimates. Fourth, acute hepatitis C cases are reported by sources of past or present medical care; consequently, some populations at risk for HCV infection (e.g., incarcerated, homeless, and uninsured persons) with limited or no access to care are likely to be underrepresented in surveillance reporting. Fifth, multiple treatment admissions by a single individual (i.e., readmissions) might have occurred within and across years and/or states and cannot be excluded from the analysis of the TEDS-A dataset. Sixth, it was not possible to analyze admission data from TEDS-A by the urbanization classification scheme because geographic identifiers represented a treatment facility’s location and not a patient’s residence. Finally, reporting requirements for substance abuse admissions to TEDS-A vary by state. This report likely does not capture all substance abuse treatment admissions within a state, but TEDS-A is estimated to include 67% of all substance abuse admissions and 83% among TEDS-A–eligible admissions in the United States.††

Although the prevalence of human immunodeficiency virus (HIV) infection among young persons who inject drugs in central Appalachia is currently low, the regional increase in cases of acute HCV infection described in this report raises concerns about the potential for an increase in HIV infections because IDU is a risk factor for both HCV and HIV infection (9). Thus, integrated health care services are needed to treat substance abuse and prevent and treat blood-borne infections deriving from illicit drug use behaviors (10). Because persons who inject drugs underutilize health services, additional efforts are urgently needed to enlist them into substance abuse treatment, ensure they are tested for HCV, and link those with HCV infection into care to receive appropriate treatment. These efforts will require further collaboration among federal partners and state and local health departments, particularly in those regions most heavily impacted, to better address the syndemic of opioid abuse and HCV infection.

## Figures and Tables

**FIGURE 1:**
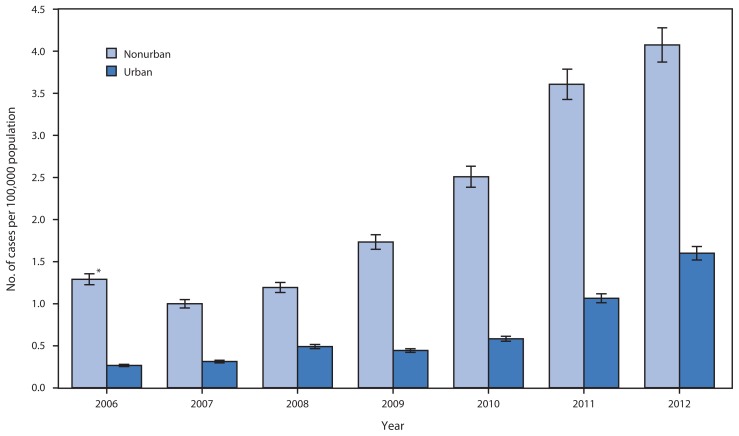
Incidence of acute hepatitis C among persons aged ≤30 years, by urbanicity and year — Kentucky, Tennessee, Virginia, and West Virginia, 2006–2012 * 95% confidence interval.

**FIGURE 2:**
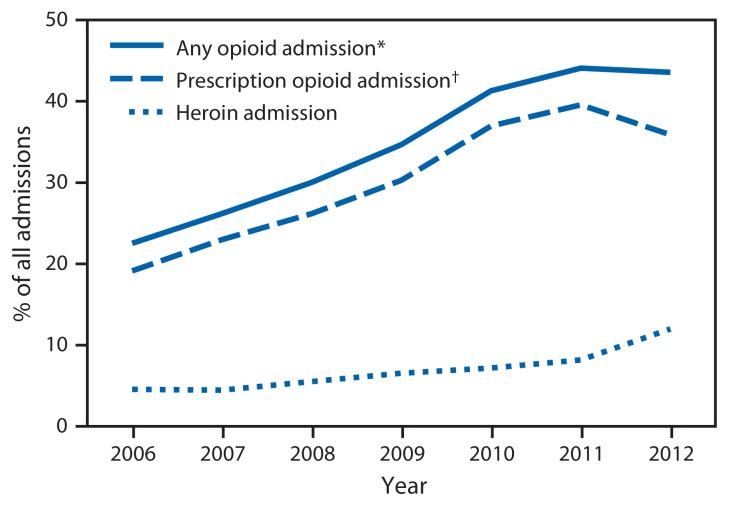
Percentage of all admissions to substance abuse treatment centers by persons aged 12–29 years (N = 217,789) attributed to the use of opioids, prescription opioids, and heroin, by year — Kentucky, Tennessee, Virginia, and West Virginia, 2006–2012 * Any opioids include heroin and prescription opioids. ^†^ Prescription opioids includes buprenorphine, codeine, hydrocodone, hydromorphone, meperidine, morphine, opium, oxycodone, pentazocine, propoxyphene, tramadol, illicitly obtained methadone, and any other drug with morphine-like effects.

**FIGURE 3:**
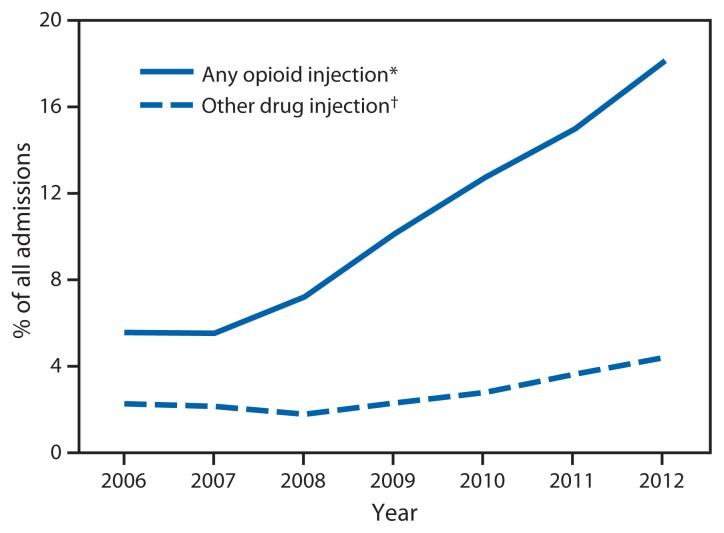
Percentage of all admissions to substance abuse treatment centers by persons aged 12–29 years (N = 217,789) attributed to the injection of opioids and other drugs, by year — Kentucky, Tennessee, Virginia, and West Virginia, 2006–2012 * Any opioids include heroin and prescription opioids. ^†^ Other drugs include cocaine/crack, alcohol, phencyclidine, other hallucinogens, methamphetamine, other amphetamines, other stimulants, benzodiazepines, other non-benzodiazepine tranquilizers, barbiturates, other non-barbiturate sedatives or hypnotics, over the counter medications, and other drugs not listed.

**TABLE:** Sociodemographic characteristics and risk factors for reported acute hepatitis C infection among adolescents and young adults aged <30 years, by urbanicity — Kentucky, Tennessee, Virginia, and West Virginia, 2006–2012

	Urban*		Nonurban†	
		
Characteristic	No.	(%)	No.	(%)
**Median age (yrs)**	**25**		**25**	
**Sex**				
Male	142	(47.2)	157	(49.8)
Female	155	(51.5)	156	(49.5)
Unknown	4	(1.3)	2	(0.6)
**Race/Ethnicity**				
Black, non-Hispanic	5	(1.7)	0	(0.0)
White, non-Hispanic	249	(82.7)	247	(78.4)
Hispanic	2	(0.7)	3	(1.0)
Other	7	(2.3)	5	(1.6)
Unknown	38	(12.6)	60	(19.0)
**Injection drug use reported** §	**99**	**(71.7)**	**95**	**(74.8)**
**Total**	**301**	**—**	**315**	**—**

* Median urban population during 2006–2012 in the four states was 6,347,762.

† Median nonurban population during 2006–2012 in the four states was 2,080,097.

§ Among cases in persons who reported any HCV risk factor (urban = 138, nonurban = 127).

## Footnotes

* From 2006 to 2012, acute hepatitis C was defined for surveillance as laboratory-confirmed infection with acute illness of discreet onset. Acute illness was considered as the presence of any sign or symptom of acute viral hepatitis plus either jaundice or elevated alanine aminotransferase >400 IU/L. In 2012, the surveillance case definition was expanded to include cases with negative HCV antibody followed by positive antibody within 6 months.

† Information available at http://wwwn.cdc.gov/nndss/conditions/hepatitis-c-acute/case-definition/2012.

§ Information available at http://www.cdc.gov/hepatitis/Statistics/2011Surveillance/PDFs/2011HepSurveillanceRpt.pdf.

¶ Information available at http://www.cdc.gov/nchs/data/series/sr_02/sr02_154.pdf.

** Information available at http://doi.org/10.3886/ICPSR25221.v9.

†† Information available at http://wwwdasis.samhsa.gov/teds95/1995_rpt.pdf.
